# Measles in Democratic Republic of Congo: an outbreak description from Katanga, 2010–2011

**DOI:** 10.1186/1471-2334-13-232

**Published:** 2013-05-22

**Authors:** Lise Grout, Andrea Minetti, Northan Hurtado, Gwenola François, Florence Fermon, Anne Chatelain, Géza Harczi, Jean de Dieu Ilunga Ngoie, Alexandra N’Goran, Francisco J Luquero, Rebecca F Grais, Klaudia Porten

**Affiliations:** 1 Epicentre, Paris, France; 2 Médecins Sans Frontières, Paris, France; 3 Médecins Sans Frontières, Lubumbashi, Democratic Republic of Congo; 4 Ministry of Health, Lubumbashi, Democratic Republic of Congo

**Keywords:** Measles, Disease outbreak, Mass vaccination, Africa south of the sahara, Control

## Abstract

**Background:**

The Democratic Republic of Congo experiences regular measles outbreaks. From September 2010, the number of suspected measles cases increased, especially in Katanga province, where Medecins sans Frontieres supported the Ministry of Health in responding to the outbreak by providing free treatment, reinforcing surveillance and implementing non-selective mass vaccination campaigns. Here, we describe the measles outbreak in Katanga province in 2010–2011 and the results of vaccine coverage surveys conducted after the mass campaigns.

**Methods:**

The surveillance system was strengthened in 28 of the 67 health zones of the province and we conducted seven vaccination coverage surveys in 2011.

**Results:**

The overall cumulative attack rate was 0.71% and the case fatality ratio was 1.40%.

The attack rate was higher in children under 4 and decreased with age. This pattern was consistent across districts and time. The number of cases aged 10 years and older barely increased during the outbreak.

**Conclusions:**

Early investigation of the age distribution of cases is a key to understanding the epidemic, and should guide the vaccination of priority age groups.

## Background

Between 2001 and 2008, measles morbidity and mortality reduced respectively by 93% and 91% in countries of the World Health Organization African region. This remarkable progress was related to an increase of measles vaccine coverage [1] and the implementation of supplementary immunization activities (SIAs). However, after reaching an historic low of 32,278 reported measles cases in 2008, the number of reported cases from WHO African region countries increased in 2009 and 2010 [2]. In 2010, 15 countries reported laboratory-confirmed measles outbreaks, up from 13 in 2009 and 9 in 2008.

The Democratic Republic of Congo (DRC) is prone to repeated measles outbreaks. DRC’s Expanded Programme on Immunization (EPI) includes one dose of measles vaccine for infants 9–11 months of age. Over the past 20 years, the estimated EPI vaccine coverage in children 1 year of age has progressively increased to 68% in 2010 according to WHO/UNICEF estimates [2]. SIAs have been implemented in DRC, including a “catch-up” campaign targeting children aged 6 months to 14 years of age between 2002 and 2006, and follow-up campaigns targeting children aged 6 to 59 months every three years [3], to provide either a second dose of measles vaccine or a second opportunity for vaccination to children not vaccinated through EPI.

The last outbreak in Katanga province occurred in 2006–2007, when more than 30,000 cases were reported, mainly from the northern districts [4]. On average, 108 suspected cases of measles were reported per month in the province in 2008, and 14 per month in 2009. A catch-up measles vaccination campaign was implemented in 2004 and a follow-up campaign in 2007, with reported administrative vaccine coverage (i.e. number of doses delivered divided by estimated number of children in the targeted age group) ranging from 80% to 142% across the 67 health zones. No SIA was implemented in 2010.

From September 2010, the number of suspected measles cases reported in the DRC increased, especially in provinces in which SIAs planned for 2010 were not implemented [3]. The first province to report cases, and the most affected, was Katanga, located the southeast of the country and bordering Zambia (where a measles outbreak occurred in 2010 [2]).

In Katanga, the non-governmental organization Médecins sans Frontières (MSF) supported the Ministry of Health (MoH) in responding to the outbreak by providing free treatment, reinforcing surveillance and implementing non-selective mass vaccination campaigns [5]. Outbreak response immunizations (ORIs) targeted children aged 6 months to 14 years in 26 health zones and children aged 6 months to 9 years in 4 helath zones of Haut-Lomami district. In total, 1,858,733 children were vaccinated with the support of MSF. In the other health zones of the province, SIAs were implemented by the MoH targeting all children aged 6 to 59 months.

Here, we describe the 2010–2011 measles outbreak in Katanga province and the results of vaccine coverage surveys conducted after the campaigns.

## Methods

### Surveillance system

National surveillance in DRC is based on the Integrated Disease Surveillance and Response strategy with measles classified as an epidemic-prone disease requiring immediate notification [6]. Early cases need to be laboratory confirmed at the National Biomedical Research Institute in Kinshasa by ELISA following WHO standards [5]. All suspected measles cases and deaths are immediately reported at health zone level, and weekly to district, provincial and national levels. WHO definitions for measles case and death are used [5].

The surveillance system was strengthened through reinforcement of the case definition and data collection, retrospective review of health registers and implementation of measles-specific registers. A dedicated team of medical staff was put in place to perform this activity. Every week, data from health facilities was reported to the health zone central office for compilation. Data entry was performed in EpiData v3.0 (Odense, Denmark).

Attack rates (ARs) by age (number of measles cases of a specific age and in a specific area divided by population figures for this age and this area) were calculated for the 28 health zones where the surveillance system was reinforced as age of cases in years was available. Estimates of population figures by health zone for 2011 were obtained from health authorities and we used the 2007 Health and demographic survey of DRC [7] for information on the age pyramid of the population.

In order to evaluate whether the age distribution of cases recorded at the very beginning of a measles outbreak (“early cases”) reflected the age distribution of all the cases recorded during the overall outbreak which should guide the choice of age groups to be targeted through vaccination campaigns, we plotted age distribution of cases from Lubumbashi city at different time-periods during the course of the epidemic. Time-periods considered for comparison were: a/from Week 1 to 50–2010 (retrospective data only, that could be considered as less consistent *a priori*); b/from Week 1–2010 to 2–2011 (retrospective data + 3 weeks of prospective data, that may correspond to the delay to decide and organise a vaccination campaign); c) from Week 1–2010 to 8–2011 (retrospective data + prospective data until the peak of the outbreak).

### Vaccine coverage surveys

We conducted seven vaccination coverage surveys in 19 health zones between February and September 2011. Surveys were conducted in all districts with mass vaccination campaign with the exception of Kolwezi district due to logistical constraints. In Haut-Lomami district, only one health zone was surveyed (Malemba-Nkulu) also due to logistical constraints. Three-stage cluster sampling was used with clusters allocated proportionally to the population of health areas and villages using estimations provided by the health authorities. In urban areas, the first compound of each cluster was selected randomly by spatial-based sampling [8]; in rural areas it was selected by spatial-based sampling if a high-definition satellite image was available, or by the EPI method if not [9]. An area was defined as urban if it included a statutory city (in DRC, all provincial capital plus 21 cities are considered as statutory). Subsequent compounds were selected by proximity (nearest compound to the left). Sample size ranged from 300 to 1368 compounds according to the design and expected vaccination coverage (from 45% to 90%). All eligible children living in selected compounds were included. Vaccination status was collected by trained surveyors from vaccination cards or through face-to-face interview with the main caregivers. Data were entered in EpiData v3.0 (Odense, Denmark) and analyzed in Stata v11 (College Station, Texas, USA). Analyses were weighted to take into account the sampling design and the varying number of children in each cluster.

### Ethical considerations

The surveillance system was reinforced in collaboration with provincial health authorities. Vaccine coverage surveys were implemented after obtaining authorization from the same authorities. Oral consent was obtained from participants and data were collected and entered anonymously. As reinforcement of the surveillance system and the coverage surveys are considered part of program monitoring, approval from MSF ethic review board was not required.

## Results

**Figure 1:**
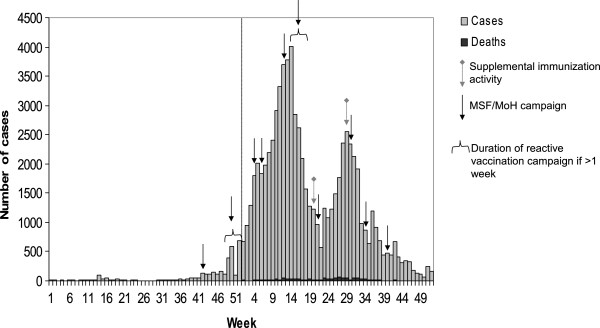
Weekly number of reported measles cases in Katanga province, Democratic Republic of Congo, 2010–2011.

**Figure 2:**
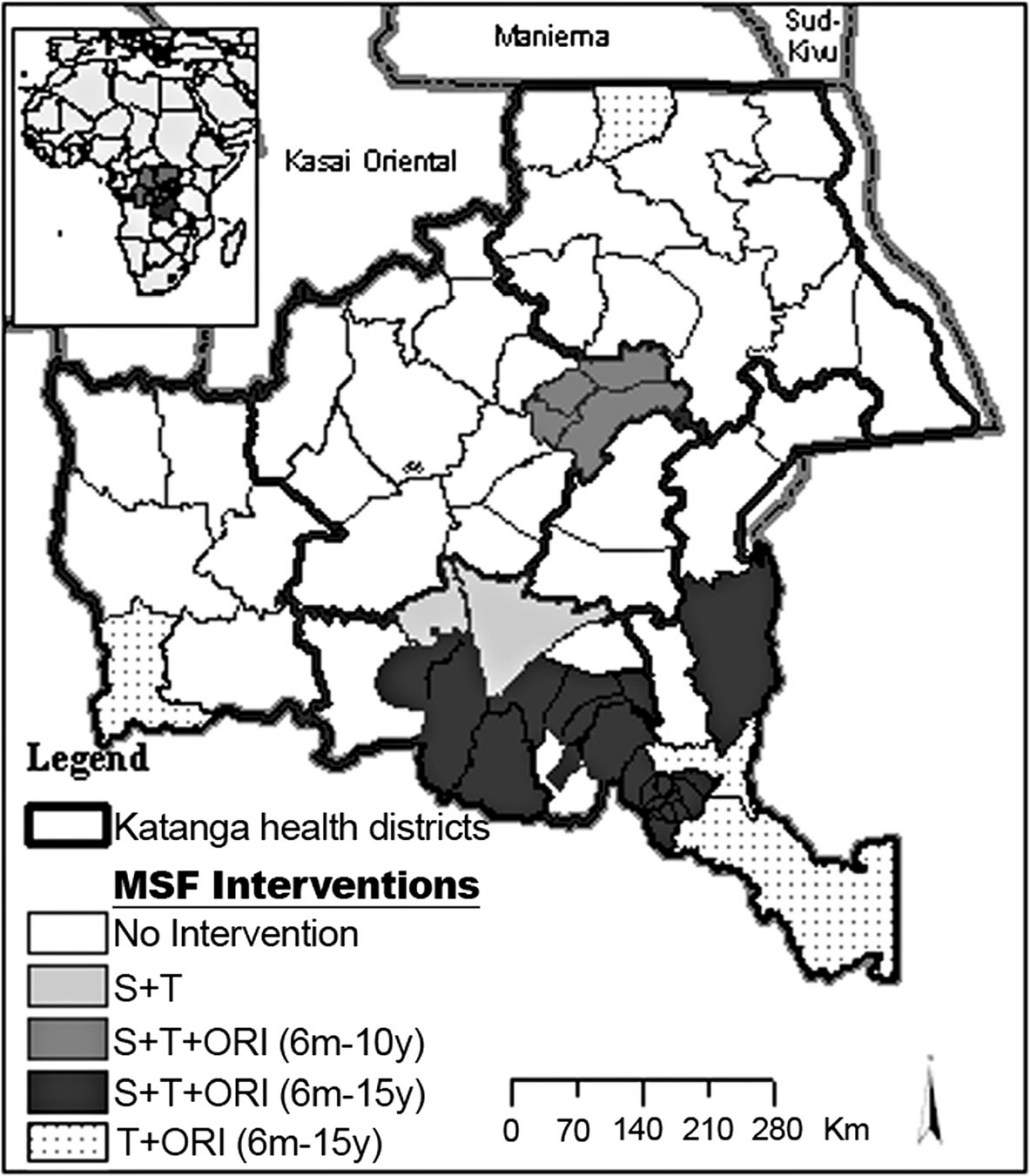
**Intervention of Médecins sans Frontières (MSF) against measles outbreak in Katanga province, Democratic Republic of Congo, in 2010 – 2011. **T = Free-of-charge treatment, S = Surveillance, ORI = Outbreak response immunization* Surveillance was also reinforced in those health zones, but data are not included in our analysis.

Sporadic suspected measles cases had been reported from several health zones of Katanga province since the beginning of 2010. The outbreak was laboratory-confirmed in Sakania health zone (Haut-Katanga district) during week 31–2010, when the first increase in measles cases was reported. Cases were subsequently reported in Kasenga health zone (Haut-Katanga district) during week 37–2010 and Dilolo and Kasaji health zones (Lualaba district) during week 38–2010. The number of measles cases reported in Likasi and Lubumbashi districts started increasing during week 42–2010, and increased substantially with the reinforcement of the prospective surveillance system in Lubumbashi and Likasi cities starting from week 50–2010 (Figure 1). The surveillance system was strengthened in 28 of the 67 health zones of the province (Figure 2).

At the provincial level, two peaks were recorded in 2011. The first peak of 3,944 cases reported in week 14–2011 corresponded to the outbreaks in Lubumbashi, Likasi and Kolwezi districts. A second peak of 2,560 cases reported in week 29–2011 corresponded to an increase in reported cases in Haut-Lomami and Tanganyika districts. Overall during the 2010–2011 epidemics, 77,241 measles cases and 1,085 deaths were reported in Katanga province. The overall cumulative AR was 0.71% and the case fatality ratio was 1.40%. The southern health zones of Katanga province were more affected than the northern ones, the most affected districts being Kolwezi and Likasi with cumulative ARs of 2.9% and 1.2% respectively.

**Figure 3:**
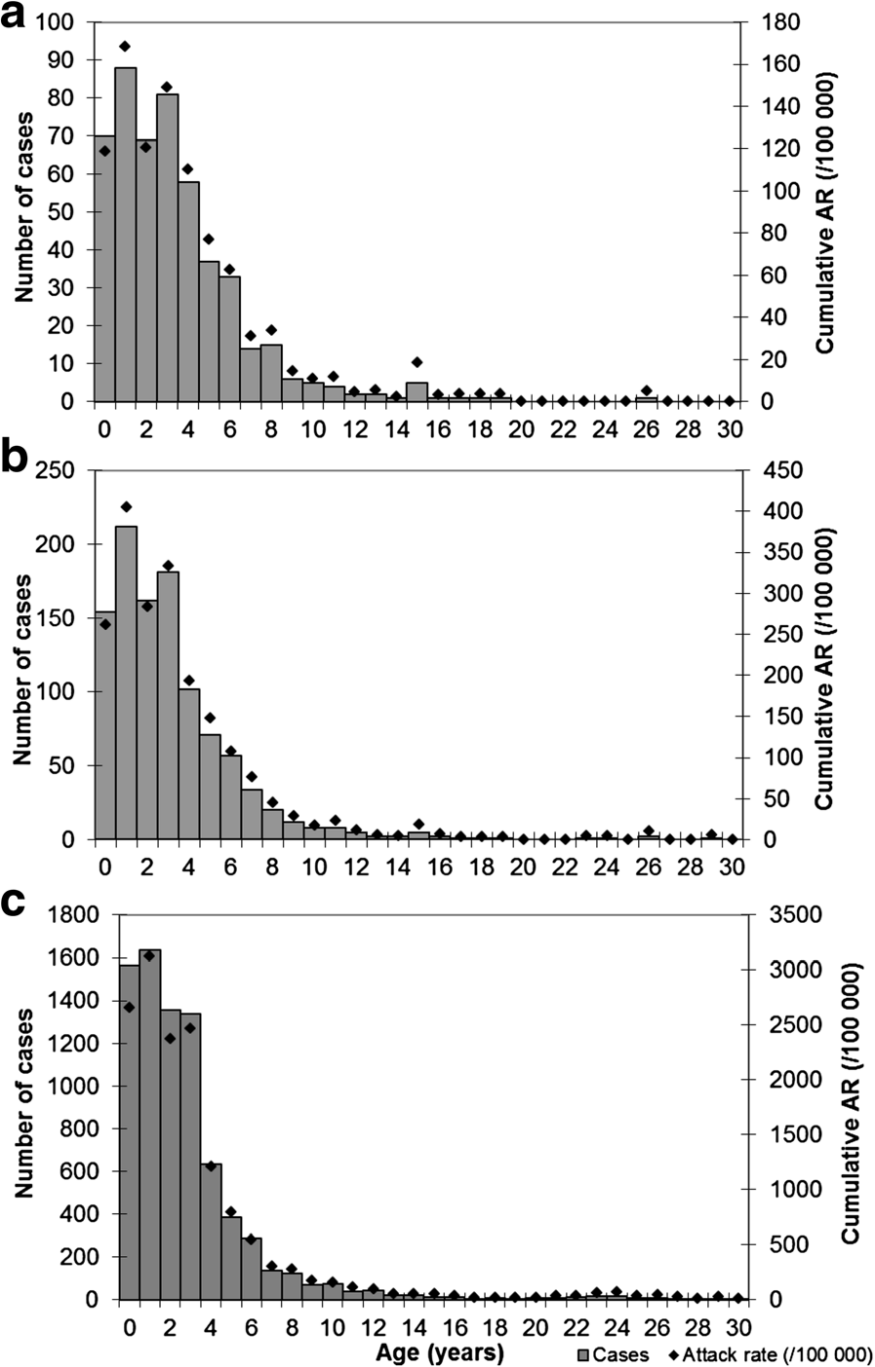
**Age distribution of cases at different time point during measles outbreak in Lubumbashi city, DRC, 2010–2011. a**)* Surveillance data from Weeks 1 to 50–2010: end of retrospective data collection (n = 496);***b**)* Surveillance data from Weeks 1–2010 to 2–2011: 3 weeks after implementation of prospective reinforced surveillance system (n = 1047);***c**)* Surveillance data from Weeks 1–2010 to 8–2011: at the outbreak peak (n = 7874).*

In the 28 health zones where surveillance was reinforced, 45,356 cases and 197 deaths were recorded, corresponding to a CFR of 0.43%. Measles cases were equally distributed between male and female (M/F: 1.0), and the median age of cases was 2.4 years (interquartile range: 1.2-4.0). Children aged under five years represented 81% of cases with the highest AR in children under 4 (4.9%) and decreasing with age. This pattern was consistently observed in all health zones where surveillance was reinforced. Age specific attack rates remained stable throughout the epidemic (Figure 3).

**Figure 4:**
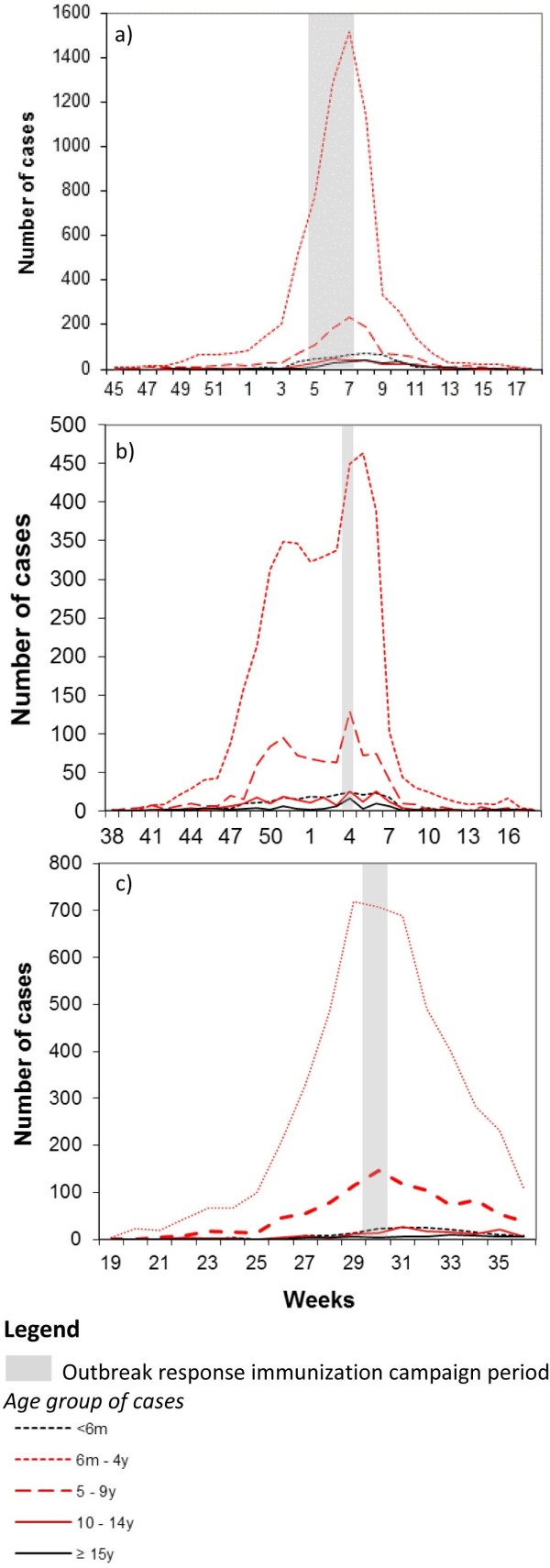
Number of cases recorded per week in (a) Likasi city, (b) Lubumbashi city and (c) Malemba-Nkulu Health zone in 2010–2011 by age group.

In the cities of Likasi and Lubumbashi, ORIs were implemented respectively during Weeks 4 and 5–2011, and targeted all children aged 6 months to 14 years. After the campaign, the number of measles cases reported decreased dramatically among children aged 6 months to 4 years (red dotted line) and 5 to 9 years (red hatched line) (Figure 4a &4b). However, the number of cases reported among children aged 10 to 14 years (red line) and ≥15 years (black line) barely increased during the outbreak and decreased slowly after the ORI.

In Malemba-Nkulu health zone (Figure 4c), the ORI conducted during week 30–2011 targeted children aged 6 months to 9 years (based on the age distribution of early cases) in order to optimize available resources. The number of reported cases among children aged 6 months to 9 years began to decrease during the week after the intervention. As observed in Likasi and Lubumbashi cities, the number of cases aged 10 to 14 years and ≥15 years barely increased during the outbreak and decreased slowly after the ORI.

**Figure 5:**
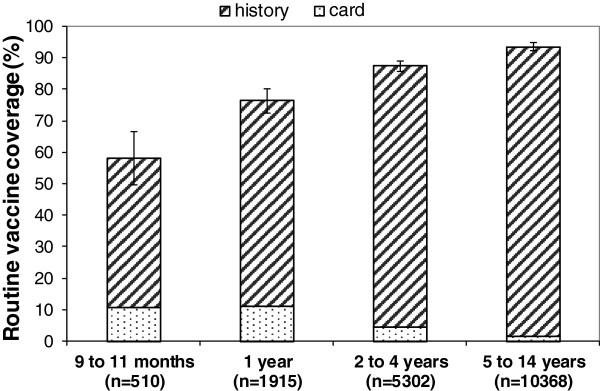
EPI measles vaccine coverage by age group estimated through seven surveys implemented in Katanga province, DRC, 2011.

During the seven vaccination coverage surveys, 4,481 compounds were visited and 18,611 children were included (Table 1). Routine (EPI) measles vaccine coverage was over 89% in Likasi city, Lubumbashi city and Kipushi health zone and below it in all the other health zones. Across all surveyed areas, coverage was lower among children aged 9 to 11 months (Figure 5). Routine coverage was higher among older children, but the upper bound of the 95% confidence interval (95% CI) remained below 90% in children aged 2 to 4 years. SIA coverage ranged from 70% to 89% across health zones surveyed.

**Table 1:** Measles vaccine coverage and their confidence interval (CI) of different immunization opportunities, by card confirmation and oral history, Katanga province, DRC, 2011

**Health district**	**Health zone**	**Type**^**1**^	**Study population**	**Number of children included**	**EPI**^**2 **^**coverage [CI 95%]**	**SIA**^**3 **^**coverage [CI 95%]**	**ORI**^**4 **^**coverage [CI 95%]**
Likasi	Kikula, Likasi and Panda	Urban	6 months - 14 years	4244	91.5% [89.1 - 93.5]	82.6% [77.6 - 86.6]	99.3% [98.9 - 99.6]
Lubumbashi	All the11 health zones of the district	Urban	6 months - 14 years	6622	91.4% [89.5 - 92.9]	89.1% [85.2 - 92.0]	98.1% [97.3 - 98.6]
Likasi	Kapolowe	Rural	6 months - 14 years	1056	83.1% [74.0 - 89.4]	81.8% [70.4 - 89.4]	97.0% [88.0 - 99.3]
Likasi	Kambove	Rural	6 months - 14 years	1223	80.0% [69.8 – 87.3]	68.2% [49.4 - 82.5]	94.7% [81.8 - 98.6]
Haut-Katanga	Kasenga	Rural	6 months - 14 years	2199	86.0% [80.4 - 90.2]	85.5% [80.4 - 89.5]	93.8% [91.0 - 95.7]
Haut-Lomami	Malemba-Nkulu	Rural	6 months - 9 years	1722	81.2% [76.4 - 85.3]	77.3% [70.6 - 82.9]	81.1% [76.5 - 84.9]
Haut-Katanga	Kipushi	Rural	6 months - 14 years	1545	92.8% [90.3 - 94.7]	84.5% [78.0 - 89.4]	97.2% [95.7 - 98.2]

^1^ An area was defined as urban if it included a statutory city (in DRC, all provincial capital plus 21 cities are considered as statutory).

^2^ Expanded Programme on Immunization (EPI) = routine vaccination activities in health facilities for children aged 9 to 11 months.

^3^ Supplementary Immunization Activities (SIA) were non-selective mass vaccination campaigns targeting children aged 6 to 59 months.

^4^ Outbreak Response Immunizations (ORI) were reactive mass vaccination campaigns targeting children aged 6 months to 14 (or 9) years.

The ORI reached a coverage above 95% in the cities of Likasi and Lubumbashi and Kipushi health zones and above 91% in Kasenga one. ORI coverage was lower in Malemba-Nkulu health zone (81.1% [95% CI: 76.5; 84.9]). There was no difference in ORI coverage across age groups.

## Discussion

The 2010–2011 measles outbreak in Katanga was one of the largest occurring during the last decade. The overall AR was higher than that reported during the previous outbreaks in Matadi or Mbuji-Mayi (DRC) in 2006 [10], comparable to that reported during the large outbreaks in N’Djamena (Chad) in 2005 and 2010 [11], but lower than those reported in Malawi in 2010 [12] and in the city of Niamey (Niger) in 2005 [13].

The AR was higher in the southern health zones of Katanga, which are densely populated and well-connected, thus facilitating disease transmission. However, the variable performance of the surveillance system should also be considered, as greater efforts were made to reinforce surveillance in southern than in northern health zones, so that the number of measles cases reported from the northern part of the province is likely underestimated.

AR was higher in children <4 years (>5%) compared with older age groups (1% in 5–9 years-old and 0.2% in 10–14 years-old). This distribution of cases across age groups observed in Katanga was in between those observed in Matadi or Mbuji-Mayi and in Malawi. In Matadi and Mbuji-Mayi [10], ARs were respectively 2.1% and 1.4% among < 5 years and 0.4% and 0.02% among children 5 to 14 years. In Malawi, AR was 2.3%, 1.1% and 0.5% among respectively <5 years, 5 to 14 years and 15 years and over [12].

The age distribution of cases was consistent across health zones and throughout the outbreak. It reflected local measles epidemiology and past control strategies. Indeed, the AR was higher among children under 4 years of age, born after the last SIA and the last measles outbreak in 2007. Children aged 4 to 8 years, eligible for the 2007 SIA and exposed to the 2007 outbreak in the north of the province, and those aged 9 years and older, eligible for the 2004 SIA (not assessed during vaccination coverage surveys) and exposed to the outbreaks in 2004 and 2007, were less affected during the 2010–11 epidemics (AR < 0.5%).

Early assessment of age-specific AR should guide the choice of age groups to be targeted through vaccination campaigns. During this outbreak, the age distribution of early cases reported in Malemba-Nkulu health zone was used to restrict the targeted age group to children aged under 10 years. Early findings from surveillance in the context of limited resources might provide important insight to aid the planning of the vaccination response by defining the priority age groups to target.

Case fatality observed was lower than reported elsewhere [14]. Deaths recorded through the surveillance system in DRC are likely underestimated as only deaths occurring in health facilities were captured. Community-based mortality surveillance may have provided a better description of measles-related mortality.

Many reasons can explain the 2010–11 epidemics in Katanga. EPI coverage among children 9–11 months was under the 90% recommended by the WHO [15], with some health zones under 80%. Routine coverage was also low among children aged 12 to 23 months, highlighting the need to enlarge the age range for providing the first dose of measles vaccine through EPI [16]. Moreover, the 2007 SIA coverage was below the 95% level recommended by WHO [15], and the SIA planned in 2010 [3] were not conducted.

Several limitations should be taken into account. First, only cases admitted to health facilities were registered in the surveillance system. As access to care is low in Katanga, especially in rural areas, the total number of measles cases may be underestimated. This was confirmed by the results of vaccine coverage surveys. Indeed, we asked if the children had symptoms since the beginning of the outbreak and could therefore estimate ARs. Estimated ARs were higher than those calculated through reinforced surveillance (estimated AR in Lubumbashi and Likasi cities were 5.6% [IC 95%: 4.9-6.4] and 6.3% [IC 95%: 4.7-8.5] respectively among the targeted children for example). Moreover, varying ARs in the different districts might partially reflect varying performance of the surveillance system as surveillance was reinforced in only 28 out of 67 health zones.

Second, we conducted vaccination coverage surveys in 19 health zones; our results cannot be extrapolated to other health zones, where coverage may be very different particularly in rural areas. Moreover, the percentage of vaccinated children ascertained by card was low, leading to potential misclassification, although previous studies have shown parental recall to be highly reliable [17]. To further minimize bias, we recalled the site of injection to the participants (measles vaccine is given in the left thigh in DRC) and the period the vaccination might have taken place. This highlights the difficulty in obtaining precise estimates of vaccination coverage in settings with weak public health infrastructure as both population figures and individual data on vaccination are not available. Future work should include serologic confirmation.

Third, the last census was carried out in 1984 in DRC. Due to uncertainty in population figures, age-specific ARs may be either under or over-estimated.

## Conclusion

The early investigation of the age distribution of measles cases is a key step in describing an epidemic, as it reflects the local epidemiological context (such as the performance of control programs and past outbreak experience) and should guide outbreak response activities. The vaccination of all children aged 6 months to 15 years might not be the most appropriate strategy in all sites. ORIs should be quickly implemented to have more impact on the outbreak. Nevertheless, the time for a detailed description of early cases should be taken to identify priority age groups to be targeted through vaccination campaigns, according to the final objective of the intervention and when resources are limited.

## Abbreviations

WHO: World Health Organization; DRC: Democratic Republic of Congo; EPI: Expanded Programme on Immunization; SIA: Supplementary Immunization Activities; VC: Vaccine coverage; MSF: non-governmental organization Médecins sans Frontières; MoH: Ministry of Health; AR: Attack rates; CFR: Case fatality ratio; IQR: Inter-quartile range; ORI: Outbreak response immunization campaigns; CI: Confidence interval.

## Competing interests

The authors declare that they have no competing interests.

## Authors’ contributions

LG implemented the study, analysed the data and drafted the manuscript; AM helped to the conception of the study, the data interpretation and to draft the manuscript; NH and FF helped to the conception of the study, the data collection and to draft the manuscript; GH, GF and AC helped to the implementation of the study, data collection and data interpretation; JDIN helped to the implementation of the study and the coordination with the Ministry of Health; AN helped in the data collection and data analysis; FJL helped to the conception of the study and to the data interpretation; RFG conceived of the study and helped to draft the manuscript. KP conceived of the study, participated in its design and coordination and helped to draft the manuscript. All authors read and approved the final manuscript.

## Pre-publication history

The pre-publication history for this paper can be accessed here:

http://www.biomedcentral.com/1471-2334/13/232/prepub
